# The cardiovascular and renal effects of glucagon-like peptide 1 receptor agonists in patients with advanced diabetic kidney disease

**DOI:** 10.1186/s12933-023-01793-9

**Published:** 2023-03-17

**Authors:** Yuan Lin, Te-Hsiung Wang, Ming-Lung Tsai, Victor Chien-Chia Wu, Chin-Ju Tseng, Ming-Shyan Lin, Yan-Rong Li, Chih-Hsiang Chang, Tien-Shin Chou, Tzu-Hsien Tsai, Ning-I Yang, Ming-Jui Hung, Tien-Hsing Chen

**Affiliations:** 1 Department of Emergency Medicine, Keelung Chang Gung Memorial Hospital, Keelung, Taiwan; 2 Chang Gung University, Taoyuan, Taiwan; 3 Department of Emergency Medicine, Medical Research Institute, Tazuke Kofukai, Kitano Hospital, Osaka, Japan; 4 Department of Primary Care and Emergency Medicine, Kyoto University Graduate School of Medicine, Kyoto, Japan; 5 Division of Cardiology, Department of Internal Medicine, New Taipei Municipal TuCheng Hospital, New Taipei City, Taiwan; 6 College of Medicine, Chang Gung University, Taoyuan, Taiwan; 7 Division of Cardiology, Department of Internal Medicine, Chang Gung Memorial Hospital, Linkou Medical Center, Taoyuan, Taiwan; 8 Department of Internal Medicine, Keelung Chang Gung Memorial Hospital, Keelung, Taiwan; 9 Division of Cardiology, Department of Internal Medicine, Chiayi Chang Gung Memorial Hospital, Chiayi, Taiwan; 10 Division of Endocrinology and Metabolism, Department of Internal Medicine , Chang Gung Memorial Hospital, Linkou Medical Center , Taoyuan, Taiwan; 11 Division of Nephrology, Department of Internal Medicine, Chang Gung Memorial Hospital, Linkou Medical Center, Taoyuan, Taiwan; 12 Division of Gastroenterology, Department of Internal Medicine, Keelung Chang Gung Memorial Hospital , Keelung, Taiwan; 13 Division of Cardiology, Department of Internal Medicine, Ditmanson Medical Foundation Chiayi Christian Hospital, Chiayi, Taiwan; 14 Division of Cardiology, Department of Internal Medicine, Keelung Chang Gung Memorial Hospital, No. 222, Maijin Rd Anle Dist., Keelung, 204 Taiwan; 15 School of Traditional Chinese Medicine, Chang Gung University, Taoyuan, Taiwan

**Keywords:** Advanced diabetic kidney disease, CV outcomes, GLP-1RA, Renal outcomes

## Abstract

**Background:**

To determine whether glucagon-like peptide 1 receptor agonists (GLP-1RAs) have cardiovascular and renal protective effects in patients with advanced diabetic kidney disease (DKD) with an estimated glomerular filtration rate (eGFR) < 30 mL/min per 1.73 m^2^.

**Methods:**

In this cohort study, patients with type 2 diabetes mellitus and eGFR < 30 mL/min per 1.73 m^2^ with a first prescription for GLP-1RAs or dipeptidyl peptidase 4 inhibitors (DPP-4is) from 2012 to 2021 (n = 125,392) were enrolled. A Cox proportional hazard model was used to assess the cardiorenal protective effects between the GLP-1RA and DDP-4i groups.

**Results:**

A total of 8922 participants [mean (SD) age 68.4 (11.5) years; 4516 (50.6%) males; GLP-1RAs, n = 759; DPP-4is, n = 8163] were eligible for this study. During a mean follow-up of 2.1 years, 78 (13%) and 204 (13.8%) patients developed composite cardiovascular events in the GLP-1RA and DPP-4i groups, respectively [hazard ratio (HR) 0.88, 95% confidence interval CI 0.68–1.13]. Composite kidney events were reported in 134 (38.2%) and 393 (44.2%) patients in the GLP-1RA and DPP-4i groups, respectively (subdistribution HR 0.72, 95% CI 0.56–0.93).

**Conclusions:**

GLP-1RAs had a neutral effect on the composite cardiovascular outcomes but reduced composite kidney events in the patients with advanced DKD compared with DPP-4is.

**Supplementary Information:**

The online version contains supplementary material available at 10.1186/s12933-023-01793-9.

## Background

Cardiovascular diseases are the leading causes of mortality in both patients with type 2 diabetes mellitus (type 2 diabetes) and chronic kidney disease (CKD) [1, 2]. Diabetic kidney disease (DKD) is also a major cause of end-stage kidney disease (ESKD) and dialysis [3]. The prevalence of diabetes mellitus (DM) is estimated to be 10.5% globally [4], and the cardiovascular mortality rate in DKD patients is more than two folds higher compared to patients with type 2 diabetes with preserved kidney function [5]. An estimated glomerular filtration rate (eGFR) < 60 mL/min per 1.73 m^2^ (unit omitted below) has been associated with a higher risk of cardiovascular death [5]. Therefore, it is important to prevent cardiovascular and kidney events in DKD patients, especially in those with poor kidney function.

Although the first-line medication for type 2 diabetes is metformin, glucagon-like peptide-1 receptor agonists (GLP-1RAs) along with sodium–glucose cotransporter 2 inhibitors (SGLT2is) are recommended for patients with established atherosclerotic cardiovascular disease (ASCVD) or multiple ASCVD risk factors [6, 7]. Cardiorenal benefits including the prevention of major adverse cardiovascular events (MACEs) and a reduction in new-onset macroalbuminuria have been demonstrated in previous landmark studies [8–13]. In addition, adding GLP-1RAs to SGLT2is in diabetic patients with heart failure has been shown to significantly reduce composite cardiovascular events [14]. Moreover, GLP-1RAs have been proposed to be a potential candidate for the prevention of obesity-related cardiovascular diseases [15]. GLP-1RAs have also been confirmed to reduce all-cause and cardiovascular mortality in patients with type 2 diabetes and to improve left ventricular diastolic function in heart failure patients; hence it is conceivable that GLP-1RAs may be beneficial for patients with advanced DKD [16, 17]. Furthermore, SGLT2is but not GLP1-RAs have been associated with a lower risk of atrial fibrillation when compared with dipeptidyl peptidase 4 inhibitors (DPP-4is) [18]. SGLT2is are recommended for DKD patients with an eGFR > 25 and urine albumin-to-creatinine ratio (UACR) > 300 mg/g creatinine, while the role of GLP-1RAs in advanced DKD patients remains controversial [6].

Research on the effect of GLP-1RAs on cardiovascular outcomes in patients with advanced DKD (eGFR < 30) is limited. Previous GLP-1RA trials have mainly excluded advanced DKD patients, and completely excluded those with ESKD. Results from the LEADER study showed favorable cardiovascular outcomes in terms of MACEs in patients receiving liraglutide with an eGFR 30–60 compared to a placebo cohort [19]. Liraglutide, lixisenatide, dulaglutide and semaglutide have been shown to reduce the development of macroalbuminuria, indicating a renal protective effect [20]. However, solid evidence of cardiovascular protective effects with lixisenatide, dulaglutide and semaglutide in advanced DKD patients is lacking. Hence, the cardiovascular impact of GLP-1RAs in patients with advanced DKD and ESKD is worth investigating.

This study enrolled type 2 diabetes patients with a first prescription for GLP-1RAs and an eGFR < 30, and compared their effect to DPP-4is. The primary outcomes were composite cardiovascular outcomes including cardiovascular death, myocardial infarction (MI) and ischemic stroke, and composite renal outcome including a decline in eGFR > 50%, progression to ESKD with dialysis, and cardiovascular death. The aim of the study was to investigate the potential cardiovascular and renal protective effects of GLP-1RAs in DKD patients with moderate to severe kidney function Additional file 1.

## Methods

### Data source

Data were acquired from Chang Gung Research Database (CGRD). The CGRD is the largest multi-institutional electronic medical record (EMR) database in Taiwan [21], including 2 medical centers and five general hospitals, and information on more than 11 million patients from 2001 to 2019.

### Patients and study design

The study cohort included patients with a first prescription for GLP-1RAs or DPP-4is from January 1, 2012 to December 31, 2021. The date of first prescription was defined as the index date. GLP-1RAs included liraglutide and dulaglutide, and DPP-4is included sitagliptin, vildagliptin, saxagliptin, and linagliptin. Patients with missing demographic data (age or sex), type 1 DM, < 40 years old, eGFR > 30, missing baseline eGFR data, and those with a follow-up period < 3 months were excluded. Patients with prescriptions for GLP-1RAs not of interest in this study, including exenatide and lixisenatide, were also excluded. The Modification of Diet in Renal Disease (MDRD) equation was used to calculate the eGFR. Patients were followed until the occurrence of an outcome (e.g., MACE), death, drug switch, or adding on another drug (e.g., GLP-1RAs to DPP-4is or GLP-1RAs added to DPP-4is) or December 31, 2021, whichever occurred first. Due to the retrospective nature of this study, no formal sample size calculation based on estimated effect size was performed.

### Covariates

The baseline characteristics included demographics, severity of DM, kidney function and stages, comorbidities, vital signs, laboratory data, and concomitant medications. Demographic data including age, sex, body mass index (BMI) and smoking were recorded. The duration of DM, baseline glycated hemoglobin (HbA1c) level, DM retinopathy and DM neuropathy were used as a proxy for the severity of DM. Kidney function and stages were categorized as an eGFR between 15 and 30, < 15, and dialysis. The baseline comorbidities included hypertension, hyperlipidemia and seven others. Charlson’s Comorbidity Index (CCI) score was also recorded. Vital signs included systolic and diastolic blood pressure and heart rate. The laboratory data included triglycerides, total cholesterol and three others. Concomitant medications were classified into glucose-lowering therapies (sulfonylurea, insulin and four others) and cardiovascular agents (antihypertension agents, lipid-lowering agents, and antiplatelet agents).

### Outcomes

Outcome measurements included clinical events and continuous outcomes. The primary cardiovascular outcome was a composition of cardiovascular death, MI, and ischemic stroke. Cardiovascular death was defined according to the standard definitions for cardiovascular and stroke endpoint events in clinical trials by the US Food and Drug Administration. The definitions of MI and ischemic stroke were acute episodes requiring hospitalization. The renal outcomes included a decline in eGFR > 50%, and progression to ESKD with dialysis. ESKD with dialysis was defined as the need for permanent dialysis regardless of hemodialysis or peritoneal dialysis. The composite renal outcome was defined as any one of a decline in eGFR > 50%, ESKD with dialysis, and cardiovascular death. The secondary outcomes were all-cause death, heart failure admission, admission due to any cause, composite major adverse limb events including newly diagnosed peripheral arterial disease, claudication, clinical limb ischemia, limb revascularization or amputation, hypoglycemia, diabetic ketoacidosis (DKA) or hyperosmolar hyperglycemic state (HHS), and infection death. The date, place and causes of death were extracted using data linked to the Taiwan Death Registry.

Continuous outcomes included systolic and diastolic blood pressure, body weight, HbA1c, eGFR, and heart rate. The continuous outcomes were extracted at baseline, and then 6, 12, 18 and 24 months of follow-up. Since the data of the patients were substantially impacted by dialysis, the continuous outcomes after dialysis at baseline or during follow-up were not analyzed.

### Statistical analysis

A propensity score matched cohort was created to compare outcomes. The propensity score was the predicted probability to be in the GLP-1RA group derived from a multivariable logistic regression model. All of the variables listed in Table 1 were included in the calculation of propensity score, except for the follow-up year which was replaced with the index date. The caliper was set as 0.2, the algorithm was greedy, and replacement was not allowed. Each patient in the GLP-1RA group was matched to 1 or more (at most 4) counterparts in the DPP-4i group. As some data on the continuous covariates were missing, single expectation–maximization imputation was performed before conducting propensity score matching. The balance of baseline characteristics between the two groups was assessed using standardized difference (STD), where an absolute STD value < 0.2 was considered to be a non-substantial difference between groups [22].

**Table 1:** Baseline characteristics of the patients before and after propensity score matching

Variable	Before matching				After matching		
	Total (n = 8922)	GLP1RA (*n* = 759)	DPP4i (*n* = 8163)	STD	GLP1RA (*n* = 602)	DPP4i (*n* = 1479)	STD
Demographics							
Age, years	68.4 ± 11.5	65.3 ± 11.3	68.7 ± 11.5	− 0.30	65.9 ± 11.5	66.3 ± 11.0	− 0.04
Male, *n* (%)	4516 (50.6)	389 (51.3)	4127 (50.6)	0.01	305 (50.7)	784 (53.01)	− 0.05
Body mass index, kg/m^2^	26.0 ± 4.5	28.1 ± 4.9	25.7 ± 4.4	0.51	27.7 ± 4.8	27.3 ± 4.5	0.10
Smoker, *n* (%)	1696 (19.0)	163 (21.5)	1,533 (18.8)	0.07	122 (20.3)	299 (20.22)	< 0.01
Severity of DM							
Duration of DM, year	6.4 ± 5.9	10.7 ± 6.2	6.0 ± 5.7	0.78	10.0 ± 6.2	8.8 ± 6.5	0.19
Baseline HbA1c, mmol/mol Baseline HbA1c, %	62 ± 21 7.8 ± 1.9	75 ± 22 9.0 ± 2.0	60 ± 19 7.6 ± 1.8	0.73	72 ± 19 8.7 ± 1.8	68 ± 24 8.4 ± 2.2	0.15
DM retinopathy, *n* (%)	2310 (25.9)	321 (42.3)	1989 (24.4)	0.39	232 (38.5)	491 (33.20)	0.11
DM neuropathy, *n* (%)	2644 (29.6)	463 (61.0)	2181 (26.7)	0.74	341 (56.6)	733 (49.56)	0.14
Kidney function and stage							
eGFR, ml/min/1.73 m^2^	19.2 ± 14.8	20.8 ± 16.0	19.0 ± 14.6	0.12	20.7 ± 15.2	20.6 ± 16.3	< 0.01
15–30, *n* (%)	4123 (46.2)	367 (48.4)	3756 (46.0)	0.05	291 (48.3)	726 (49.09)	− 0.01
< 15, *n* (%)	1532 (17.2)	68 (9.0)	1464 (17.9)	− 0.27	60 (10.0)	163 (11.02)	− 0.03
Dialysis, *n* (%)	3267 (36.6)	324 (42.7)	2943 (36.1)	0.14	251 (41.7)	590 (39.89)	0.04
Baseline comorbidity							
Hypertension, *n* (%)	7862 (88.1)	717 (94.5)	7145 (87.5)	0.24	563 (93.5)	1364 (92.22)	0.05
Hyperlipidemia, *n* (%)	4560 (51.1)	586 (77.2)	3974 (48.7)	0.62	442 (73.4)	982 (66.40)	0.15
Coronary heart disease, *n* (%)	2751 (30.8)	323 (42.6)	2428 (29.7)	0.27	237 (39.4)	536 (36.24)	0.06
Heart failure hospitalization, *n* (%)	1438 (16.1)	160 (21.1)	1278 (15.7)	0.14	120 (19.9)	280 (18.93)	0.03
Coronary intervention, *n* (%)	1027 (11.5)	158 (20.8)	869 (10.6)	0.28	104 (17.3)	241 (16.29)	0.03
Ischemic stroke, *n* (%)	1023 (11.5)	100 (13.2)	923 (11.3)	0.06	79 (13.1)	166 (11.22)	0.06
Myocardial infarction, *n* (%)	806 (9.0)	124 (16.3)	682 (8.4)	0.24	84 (14.0)	184 (12.44)	0.04
Atrial fibrillation, *n* (%)	708 (7.9)	57 (7.5)	651 (8.0)	− 0.02	44 (7.3)	117 (7.91)	− 0.02
Peripheral arterial disease, *n* (%)	1009 (11.3)	121 (15.9)	888 (10.9)	0.15	90 (15.0)	209 (14.13)	0.02
Charlson’s Comorbidity Index score	5.5 ± 2.8	6.6 ± 2.7	5.4 ± 2.8	0.42	6.5 ± 2.7	6.2 ± 2.8	0.11
Vital sign							
Systolic blood pressure, mmHg	141.9 ± 25.5	142.5 ± 24.4	141.8 ± 25.6	0.03	142.1 ± 23.6	142.1 ± 23.9	< 0.01
Diastolic blood pressure, mmHg	73.9 ± 13.6	74.7 ± 15.6	73.8 ± 13.4	0.06	74.5 ± 15.7	74.4 ± 13.0	0.01
Heart rate, beat/min	82.2 ± 14.8	82.8 ± 13.8	82.1 ± 14.9	0.05	83.0 ± 13.7	82.9 ± 13.9	0.01
Biochemistry data							
Triglyceride, mg/dL	184.6 ± 133.2	224.9 ± 162.6	180.5 ± 129.2	0.30	211.9 ± 144.4	201.0 ± 143.6	0.08
Total cholesterol, mg/dL	173.4 ± 50.1	172.5 ± 51.1	173.5 ± 50.0	− 0.02	171.8 ± 46.7	172.4 ± 48.1	− 0.01
High-Density Lipoprotein, mg/dL	40.8 ± 13.4	39.8 ± 13.2	40.9 ± 13.5	− 0.08	40.4 ± 12.2	40.6 ± 12.7	− 0.01
Low-density lipoprotein, mg/dL	74.5 ± 14.1	72.7 ± 14.9	74.7 ± 14.0	− 0.14	73.1 ± 13.5	73.5 ± 14.2	− 0.03
UACR, mg/g	974 [130, 3006]	1036 [149, 2791]	967 [129, 3014]	NA	2138 [769, 3427]	2221 [898, 3400]	NA
Concomitant glucose lowering therapies							
Sulfonylurea, *n* (%)	5349 (60.0)	479 (63.1)	4870 (59.7)	0.07	377 (62.6)	904 (61.12)	0.03
Thiazolidinedione, *n* (%)	757 (8.5)	180 (23.7)	577 (7.1)	0.47	121 (20.1)	217 (14.67)	0.14
Glinide, *n* (%)	1930 (21.6)	140 (18.4)	1790 (21.9)	− 0.09	117 (19.4)	280 (18.93)	0.01
Alpha glucosidase, *n* (%)	1193 (13.4)	152 (20.0)	1041 (12.8)	0.20	116 (19.3)	254 (17.17)	0.05
SGLT2i, *n* (%)	178 (2.0)	64 (8.4)	114 (1.40)	0.33	44 (7.3)	86 (5.81)	0.06
Insulin, *n* (%)	2792 (31.3)	310 (40.8)	2482 (30.4)	0.22	237 (39.4)	545 (36.85)	0.05
Concomitant cardiovascular agents							
ACEi/ARB, *n* (%)	5485 (61.5)	485 (63.9)	5000 (61.3)	0.05	384 (63.8)	936 (63.29)	0.01
Beta-blocker, *n* (%)	3128 (35.1)	321 (42.3)	2807 (34.4)	0.16	252 (41.9)	577 (39.01)	0.06
DCCB, *n* (%)	6050 (67.8)	505 (66.5)	5545 (67.9)	− 0.03	397 (65.9)	998 (67.48)	− 0.03
Thiazide, *n* (%)	352 (3.9)	32 (4.2)	320 (3.9)	0.01	22 (3.7)	55 (3.72)	< 0.01
MRA, *n* (%)	630 (7.1)	59 (7.8)	571 (7.0)	0.03	47 (7.8)	110 (7.44)	0.01
Nitrates, *n* (%)	2249 (25.2)	204 (26.9)	2045 (25.1)	0.04	154 (25.6)	393 (26.57)	− 0.02
Vasodilator, *n* (%)	950 (10.6)	70 (9.2)	880 (10.8)	− 0.05	62 (10.3)	157 (10.62)	− 0.01
Statins, *n* (%)	4372 (49.0)	566 (74.6)	3806 (46.6)	0.60	428 (71.1)	1000 (67.61)	0.08
Fibrates, *n* (%)	647 (7.3)	96 (12.6)	551 (6.7)	0.20	65 (10.8)	139 (9.40)	0.05
Aspirin, *n* (%)	3007 (33.7)	303 (39.9)	2704 (33.1)	0.14	232 (38.5)	566 (38.27)	0.01
Clopidogrel/Ticagrelor/Prasugrel, *n* (%)	1662 (18.6)	179 (23.6)	1483 (18.2)	0.13	136 (22.6)	332 (22.45)	< 0.01
Follow-up, year	3.2 ± 2.5	2.1 ± 1.8	3.3 ± 2.6	− 0.53	2.2 ± 2.0	2.1 ± 2.1	0.06

Data are presented as frequency (percentage), mean ± standard deviation or median [25th, 75th percentile]

*GLP1RA* glucagon-like peptide-1 receptor agonist; *DPP4i* dipeptidyl peptidase 4 inhibitor; *DM* diabetes mellitus; *HbA1c* glycated hemoglobin; *eGFR* estimated glomerular filtration rate; *UACR* urine albumin-to-creatinine ratio; *SGLT2i* sodium-glucose cotransporter 2 inhibitor; *ACEi* angiotensin converting enzyme inhibitor; *ARB* angiotensin receptor blocker; *DCCB* dihydropyridine calcium channel blocker; *MRA* mineralocorticoid receptor antagonist

The risk of a fatal outcome (e.g., cardiovascular death, all-cause death) between groups was compared using a Cox proportional hazard model. The incidence of nonfatal clinical events (e.g., MI, eGFR decline > 50%) between groups was compared using the Fine and Gray subdistribution hazard model which considered all-cause death during follow-up as a competing risk. Post hoc subgroup analysis of composite cardiovascular outcome and new-onset dialysis was further conducted. The selected subgroup variables were age (< 65 vs. ≥ 65 years), sex, duration of DM (< 10 vs. ≥ 10 years) and ten others. Changes in the continuous outcomes from baseline to follow-up measurements between groups were compared using a linear mixed model, with the random intercept and slope. The duration from baseline to dialysis during follow-up was compared between the two groups using the Mann–Whitney U-test. The cause of death between groups was compared using the chi-square test. Statistical analyses were performed using SAS version 9.4 (SAS Institute, Cary, NC, USA). All statistical tests were 2-sided, and a *P* value < 0.05 was considered significant.

## Results

### Patient inclusion

**Fig. 1:**
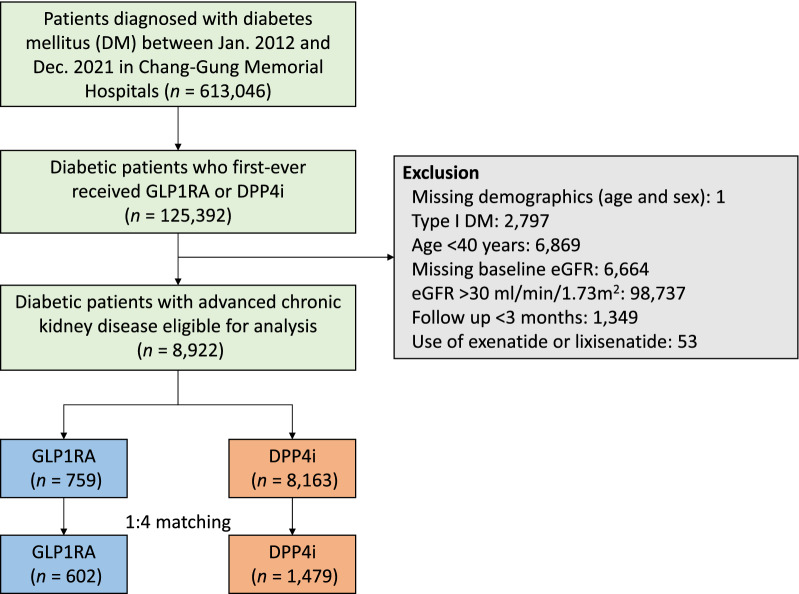
Selection of Study Patients. *GLP-1RA* glucagon-like peptide 1 receptor agonist; *DPP-4i* dipeptidyl peptidase 4 inhibitor; *DM* diabetes mellitus; *eGFR* estimated glomerular filtration rate

This study enrolled 125,392 patients with a first prescription for GLP-1RAs or DPP-4is between January 1, 2012 and December 31, 2021 (Table S1, S2). According to the exclusion criteria, a total of 759 GLP-1RA users and 8,163 DPP-4i users were eligible for analysis (Fig. 1). In the matched cohort, 212, 117, 59 and 214 patients in the GLP-1RA group were matched to 1, 2, 3 and 4 counterparts in the DPP-4i group, respectively, resulting in a total of 1479 patients in the DPP-4i group and 602 in the GLP-1RA group.

### Demographic data

The mean age of the participants was 68.4 ± 11.5 years, and 4,516 (50.6%) were male (Table 1). The mean duration of DM was 6.4 ± 5.9 years, and the baseline HbA1c was 62 ± 21 mmol/mol (7.8 ± 1.9%). Compared to the patients with DPP-4is, those with GLP-1RAs were younger, had a higher BMI, longer duration of DM, higher baseline HbA1c level, less CKD stage 5 (eGFR < 15), higher prevalence of DM retinopathy and neuropathy, hypertension, hyperlipidemia, coronary heart disease, coronary intervention and MI, greater CCI scores, higher triglyceride level, and were more likely to take thiazolidinedione, alpha glucosidase, SGLT2is, insulin, statins and fibrates (absolute SD values > 0.2). After matching, there were no significant differences in the baseline characteristics between groups (absolute SD values < 0.2).

### Clinical events

**Fig. 2:**
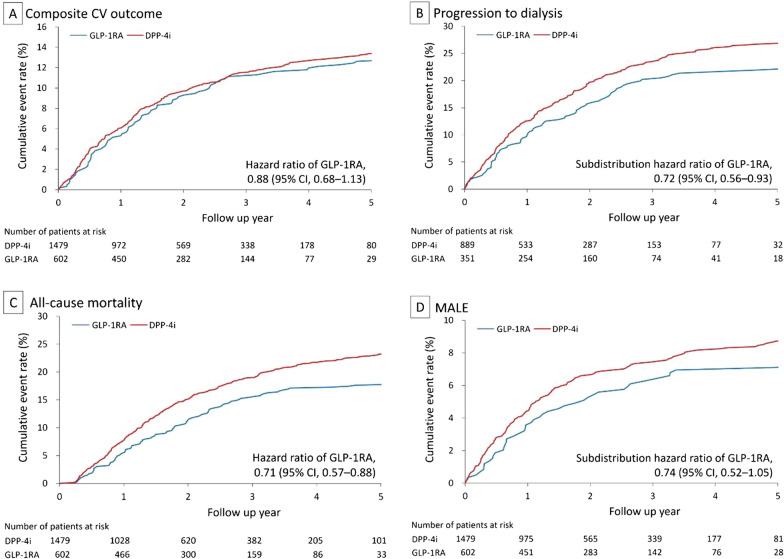
Cumulative Event Rate of Primary CV outcomes, Progression to Dialysis, All-cause Mortality, and MALEs. GLP-1RAs had a neutral effect on composite CV outcomes, but delayed progression to dialysis, and reduced all-cause mortality and MALEs compared with DDP-4is. *CV* cardiovascular; *GLP-1RA* glucagon-like peptide 1 receptor agonist; *DPP-4i* dipeptidyl peptidase 4 inhibitor; *MALEs* major adverse limb events

The mean follow-up in the matched cohort was 2.1 years (standard deviation = 2.1 years). The results showed that the risk of composite cardiovascular outcome was not significantly different between the GLP-1RA and DPP-4i groups (13% vs. 13.8%, hazard ratio [HR] 0.88, 95% confidence interval CI 0.68–1.13) (Fig. 2A). The risks of each component of the composite cardiovascular outcome were also not significantly different between the two groups, including MI, ischemic stroke and cardiovascular death. With regards to the renal outcomes, the GLP-1RA group showed a greater protective effect than the DPP-4i group, including progression to ESKD with dialysis (23.4% vs. 27.45%, subdistribution HR [SHR] 0.72, 95% CI 0.56–0.93) (Fig. 2B), decline in eGFR > 50%, and the composite renal outcomes. The median duration to new-onset dialysis was significantly longer in the GLP-1RA group (median: 1.9 years, interquartile range: 0.9–2.8 years) than in the DPP-4i group (median: 1.3 years, interquartile range: 0.6–2.4 years) (Fig. S1A).

For the secondary outcomes, the risks of all-cause death (18.4% vs. 25.1%, HR 0.71, 95% CI 0.57–0.88) (Fig. 2C) and all-cause readmission were significantly lower in the GLP-1RA group (Table 2). In addition, the risk of composite major adverse limb events in the GLP-1RA group was borderline significantly lower than that in the DPP-4i group (Fig. 2D). The common causes of death in the advanced DKD patients included malignancy, infection, CV diseases, DM, and kidney disease. There were no significant differences between the GLP-1RA and DPP-4i groups. The other causes of death were significantly lower in the GLP-1RA group (Fig. S1B).

**Table 2:** Clinical events of the patients in the propensity score matched cohort

Outcome (HR or SHR)	GLP1RA (n = 602)		DPP4i (n = 1479)		HR/SHR (95% CI) of GLP1RA	*P*
	*n* (%)	Incidence (95% CI)^a^	*n* (%)	Incidence (95% CI)^a^		
Cardiovascular outcome						
Composite CV outcome^b^ (HR)	78 (13.0)	6.0 (4.7–7.4)	204 (13.79)	6.9 (6.0–7.9)	0.88 (0.68–1.13)	0.308
Cardiovascular death (HR)	34 (5.6)	2.5 (1.7–3.4)	81 (5.5)	2.6 (2.0–3.1)	0.97 (0.66–1.44)	0.894
Myocardial infarction (SHR)	41 (6.8)	3.1 (2.2–4.1)	99 (6.7)	3.3 (2.7–4.0)	0.96 (0.67–1.38)	0.843
Ischemic stroke (SHR)	20 (3.3)	1.5 (0.9–2.18)	52 (3.5)	1.7 (1.2–2.15)	0.91 (0.54–1.52)	0.708
Renal outcome (SHR)						
eGFR decline > 50%	113 (32.2)	17.1 (14.0–20.3)	319 (35.9)	22.4 (20.0–24.9)	0.74 (0.60–0.91)	0.005
Progression to ESKD with dialysis	82 (23.4)	11.4 (8.9–13.9)	244 (27.5)	15.9 (13.9–17.9)	0.72 (0.56–0.93)	0.010
Composite renal outcome^c^	134 (38.2)	23.2 (19.3–27.1)	393 (44.2)	34.3 (30.9–37.7)	0.75 (0.61–0.93)	0.009
Secondary outcome						
All-cause death (HR)	111 (18.4)	8.3 (6.8–9.9)	371 (25.1)	12.0 (10.8–13.2)	0.71 (0.57–0.88)	0.002
Heart failure admission (SHR)	77 (12.8)	6.2 (4.8–7.5)	222 (15.1)	8.0 (6.9–9.0)	0.80 (0.61–1.05)	0.102
Admission due to any cause (SHR)	343 (57.0)	42.5 (38.0–47.0)	906 (61.3)	54.7 (51.2–58.3)	0.81 (0.71–0.91)	0.001
Composite MALE outcome^d^ (SHR)	42 (7.0)	3.3 (2.3–4.3)	132 (8.9)	4.5 (3.7–5.2)	0.74 (0.52–1.05)	0.094
Hypoglycemia (SHR)	45 (7.5)	3.5 (2.5–4.5)	117 (7.9)	4.0 (3.3–4.7)	0.89 (0.64–1.23)	0.479
DKA/HHS (SHR)	83 (13.8)	6.8 (5.3–8.2)	170 (11.5)	6.0 (5.1–6.9)	1.15 (0.88–1.51)	0.315

Data are presented as frequency (percentage)

*GLP1RA* glucagon-like peptide-1 receptor agonist; *DPP4i* dipeptidyl peptidase 4 inhibitor; *HR* hazard ratio; *SHR* subdistribution hazard ratio; *CI* confidence interval; *eGFR* estimated glomerular filtration rate; *ESKD* end-stage kidney disease; *MALE* major adverse limb event; *DKA* diabetic ketoacidosis; *HHS* hyperglycemic hyperosmolar state

^a^Number of events per 100 person-years

^b^Composite of cardiovascular death, myocardial infarction or ischemic stroke

^c^Composite of eGFR decline > 50%, progression to ESKD with dialysis or cardiovascular death

^d^Composite of newly-diagnosed peripheral arterial disease, claudication, clinical limb ischemia, limb revascularization or amputation

## Discussion

In this cohort study of patients with advanced DKD, we evaluated the associations between cardiovascular and kidney outcomes in patients with GLP-1RAs versus DPP-4is. GLP-1RAs and DPP-4is have been compared in patients with fair kidney function in previous studies, which have reported a decrease in HbA1c [23–25] and reduction in body weight [25]. Compared with the DPP-4i group, the GLP-1RA group exhibited modest benefits in terms of the composite cardiovascular outcome including cardiovascular death, MI, and ischemic stroke. In addition, the GLP-1RAs had a more favorable renal protective effect than DPP-4is in terms of a decline in eGFR > 50% and progression to ESKD with dialysis. Moreover, the GLP-1RA group had a lower rate of all-cause death and admission due to any cause. Taken together, our findings showed that the use of GLP-1RAs in type 2 diabetes patients with advanced DKD resulted in a neutral cardiovascular effect, better kidney function preservation, and lower mortality.

### Cardiovascular outcomes

GLP-1RAs have been associated with a significant reduction in composite cardiovascular outcomes in type 2 diabetes patients with relatively fair kidney function (eGFR > 30) [9, 10, 26], whereas neutral composite cardiovascular outcomes have been reported in patients with poor kidney function (eGFR < 30) [9, 10, 27]. However, these previous studies were mainly based on subgroup analysis or included only a limited sample size. Our study focused on DKD patients with an eGFR < 30 to evaluate the exact effect of GLP-1RAs on cardiovascular outcomes. We found that GLP-1RAs did not significantly improve the composite cardiovascular outcome. The pathophysiological mechanism between DKD and cardiovascular diseases is complex and multifactorial. Increased rates of cardiovascular events or death have been associated with deteriorating kidney function [28]. The SUSTAIN-6 study reported that the reduction in composite cardiovascular events was mainly attributed to nonfatal stroke [10]. In addition, the patients with advanced DKD had more resistant or difficult-to-control hypertension, which is also a major risk factor for ischemic stroke. In addition, GLP-1RAs act through several brain receptors, including the arcuate nucleus, paraventricular nucleus and subfornical organ, leading to reduced appetite, oxidative stress and inflammation [29]. These histopathological changes can contribute to mitochondrial dysfunction, subsequently leading to oxidative stress and inflammation [29], which may increase the risk of stroke in CKD patients. Other factors associated with stroke in CKD patients include alterations in cardiac output, platelet function, regional cerebral perfusion, accelerated systemic atherosclerosis, altered blood brain barrier, and disordered neurovascular coupling [30]. These CKD-related factors may have precipitated stroke and diminished the protective effect of GLP-1RAs in our study cohort, which may explain the insignificant effect on cardiovascular outcomes.

### Renal outcomes

In contrast, a significant renal protective effect was found in the GLP-1RA group compared to the DPP-4is group with regards to a decline in eGFR > 50% and ESKD progression to dialysis. The time to dialysis initiation was 6 months later in the GLP-1RA group than in the DPP-4is group. There are multiple hypotheses for the kidney protective effect of GLP-1RAs, however the mechanism remains unclear. Possible indirect factors include appropriate body weight maintenance and glycemic control, while direct factors target the kidneys. GLP-1RAs have several extra-pancreatic functions, including reducing oxidative stress-induced autophagy and endothelial dysfunction [31]. GLP-1RAs have also been shown to reduce albuminuria and glomerular sclerosis by suppressing oxidative stress and local inflammation [32]. In addition, natriuresis and potential renal protection have been proposed via sodium–hydrogen exchanger 3 (NHE3) in healthy and obese male participants [33]. A previous GLP-1RA trial in patients with relatively fair kidney function demonstrated notable renal protective effects. The LEADER study (liraglutide, eGFR > 30) revealed benefits on composite renal outcome, mostly due to a reduction in new-onset persistent macroalbuminuria [12], which is a known predictive factor of kidney-related outcomes [34]. The ELIXA study (lixisenatide, eGFR > 30) showed a reduction in UACR and lower risk of new-onset macroalbuminuria [13], and the REWIND study (dulaglutide, eGFR > 15) reported improvements in new macroalbuminuria, a sustained decline in eGFR of 30% or more, or chronic renal replacement therapy [8]. The SUSTAIN-6 study (semaglutide, eGFR > 30) reported the amelioration of persistent macroalbuminuria, doubling of serum creatinine and creatinine clearance < 45 mL/min, or continuous renal replacement therapy [10]. Nevertheless, these studies basically excluded patients with advanced CKD, especially those with an eGFR < 30. Moreover, GLP-1RA acts on the kidneys to increase renal plasma flow and glomerular filtration rate via GLP-1 receptors, and the effect of GLP-1RAs may fluctuate with different pathological status of the kidneys [35]. Thus, the actual renal protective effect of GLP-1RAs in patients with advanced DKD remains inconclusive. Our study provides evidence of a protective effect on kidney function and delay in the timing of dialysis with GLP-1RA treatment, even in patients with CKD stage 4 or 5 and type 2 diabetes.

### Secondary outcomes

We also found a significant reduction in all-cause death and admission due to any cause in the GLP-1RA cohort, which is compatible with a previous study on patients with ESKD [36]. Previous studies have generally emphasized admission due to heart failure, however the LEADER [9], ELIXA [37], REWIND [8], SUSTAIN-6 [10], PIONEER-6 (oral semaglutide) [38], EXSCEL (exenatide) [39], and Harmony (albiglutide) [40] studies all reported no significant difference in heart failure admission. The same trend was also revealed in our investigation. In addition, the LEADER, EXSCEL, and PIONEER-6 studies indicated that patients with GLP-1RAs had a lower rate of all-cause death, which is compatible with our findings [9, 38, 39]. Our GLP1-RA group did not show superiority in the composite cardiovascular outcome or cardiovascular death compared to the DDP4i group. Therefore, the decrease in all-cause death cannot be explained by heart failure admission or cardiovascular events. It is possible that the reason for the lower all-cause death rate may be related to renal death or infection death. A Scandinavian register-based cohort study demonstrated a significantly lower admission rate for kidney events in patients receiving GLP-1RAs [41]. We also demonstrated the renal protective effect of GLP-1RAs. Furthermore, GLP-1RAs have been shown to modulate sepsis. Lipopolysaccharide-induced endotoxemia, endotoxic shock, vascular dysfunction, and inflammatory markers were ameliorated by liraglutide in rat model [42]. The anti-inflammatory function of GLP1-RAs was suggested to be through the inhibition of tumor necrosis factor alpha (TNFα) and decreases in vascular cell adhesion protein 1 (VCAM-1), intercellular adhesion molecules 1 (ICAM-1) and E-selectin expression in an animal sepsis model [43]. In addition, septic acute kidney injury has been shown to induce the expression of GLP-1 receptors in renal tubules to reduce kidney injury [44]. GLP-1 receptors are expressed in several organs including the pancreas, kidneys and heart [45]. GLP-1RAs modulate not only glycemic control but also inflammation. These sophisticated interactions of GLP-1RAs including the decrease in renal and infection events may explain the decrease in all-cause death and admission due to any cause.

### Limitations

Although this study is based on real-world data on outcomes of patients with advanced DKD receiving GLP-1RAs, there are several limitations. First, we cannot infer causal associations between GLP-1RAs and cardiovascular or kidney outcomes due to the retrospective observational design of this study. Nevertheless, we enrolled patients who received GLP-1RAs and DPP-4is and evaluated the same parameters and outcomes in both groups. Therefore, the causal relationship should be relatively valid in this study. Second, background heterogeneity existed in the GLP-1RA and DPP-4i cohorts. The GLP-1RA users usually had a longer DM duration, more complications, and a refractory tendency to antiglycemic agents. These differences may have interfered with the outcomes; however, we mitigated sampling bias using propensity score matching to balance covariates including DM duration, DM complications, drug categories, and laboratory data. Therefore, we believe that the study outcomes should not be influenced by heterogeneity. Third, it is difficult to avoid coding errors in database research. We diminished possible miscoding by pairing diagnostic code and drug registration data. For instance, hypertension was defined as patients receiving antihypertensive agents and a diagnosis of hypertension, and similar definitions were also applied to other diseases. We also defined kidney function using direct eGFR data rather than CKD stage diagnosis code, which may have been coded inappropriately. In addition, the outcome measurements including ischemic stroke and MI required admission records. Therefore, disease miscoding in this study should be limited. Fourth, the GLP-1RAs in this study only included the human GLP-1-like analogues liraglutide and dulaglutide. Semaglutide was not included because few patients used this drug as it was relatively new in Taiwan during the enrollment period. We excluded exendin-4-like analogues such as exenatide and lixisenatide because they are different drug subcategories. Although the outcomes were limited to liraglutide and dulaglutide, the results should be robust and homogenous. Finally, we cannot ensure medication compliance in each patient, which is a common limitation in real-word observational studies. However, the National Health Insurance Administration in Taiwan created the Diabetic Shared Care Program (DSCP) to ensure that diabetic patients receive standard care in Taiwan. The DSCP team includes physicians, nurses, nutritionists and pharmacists who receive standard care courses to provide integrated care. This approach should increase the medication adherence of diabetic patients in Taiwan.

## Conclusions

GLP-1RAs had no influence on the composite cardiovascular outcomes but reduced composite kidney events including a decline in eGFR > 50% and progression to ESKD with dialysis, all-cause mortality, and admission in patients with advanced DKD (eGFR < 30) compared with DPP-4is.

## Supplementary Information

**Additional file 1: Figure S1**. Time to Dialysis Distribution and Causes of Death. **Table S1.** Number of patients with advanced chronic kidney disease with prescription of GLP1RA and DPP4i. **Table S2.** Number of patients receiving dialysis with prescription of GLP1RA and DPP4i.

## Data Availability

The datasets used and/or analyzed during the current study are available from the corresponding author on reasonable request.

## Abbreviations

ASCVD: Atherosclerotic cardiovascular disease
BMI: Body mass index
CCI: Charlson’s Comorbidity Index
CGRD: Chang Gung Research Database
CI: Confidence interval
CKD: Chronic kidney disease
DKA: Diabetic ketoacidosis
DKD: Diabetic kidney disease
DM: Diabetes mellitus
DPP-4is: Dipeptidyl peptidase 4 inhibitors
DSCP: Diabetic Shared Care Program
eGFR: Estimated glomerular filtration rate
EMR: Electronic medical record
ESKD: End-stage kidney disease
GLP-1RAs: Glucagon-like peptide 1 receptor agonists
HbA1c: Glycated hemoglobin
HHS: Hyperosmolar hyperglycemic state
HR: Hazard ratio
ICAM-1: Intercellular adhesion molecules 1
MACEs: Major adverse cardiovascular events
MDRD: Modification of diet in renal disease
MI: Myocardial infarction
NHE3: Sodium–hydrogen exchanger 3
SGLT2is: Sodium–glucose cotransporter 2 inhibitors
SHR: Subdistribution hazard ratio
STD: Standardized difference
TNFα: Tumor necrosis factor alpha
UACR: Urine albumin-to-creatinine ratio
VCAM-1: Vascular cell adhesion protein 1
