# Supplementary material for: The cardiovascular and renal effects of glucagon-like peptide 1 receptor agonists in patients with advanced diabetic kidney disease
Source: Cardiovasc Diabetol. 2023 Mar 17;22:60. doi: 10.1186/s12933-023-01793-9 (PMC10024371; doi:10.1186/s12933-023-01793-9)
Supplement: Supplementary file 1 — Additional file 1: Figure S1. Time to Dialysis Distribution and Causes of Death. Table S1. Number of patients with advanced chronic kidney disease with prescription of GLP1RA and DPP4i. Table S2. Number of patients receiving dialysis with prescription of GLP1RA and DPP4i. [file 12933_2023_1793_MOESM1_ESM.docx]

**Figure S1. Time to Dialysis Distribution and Causes of Death**


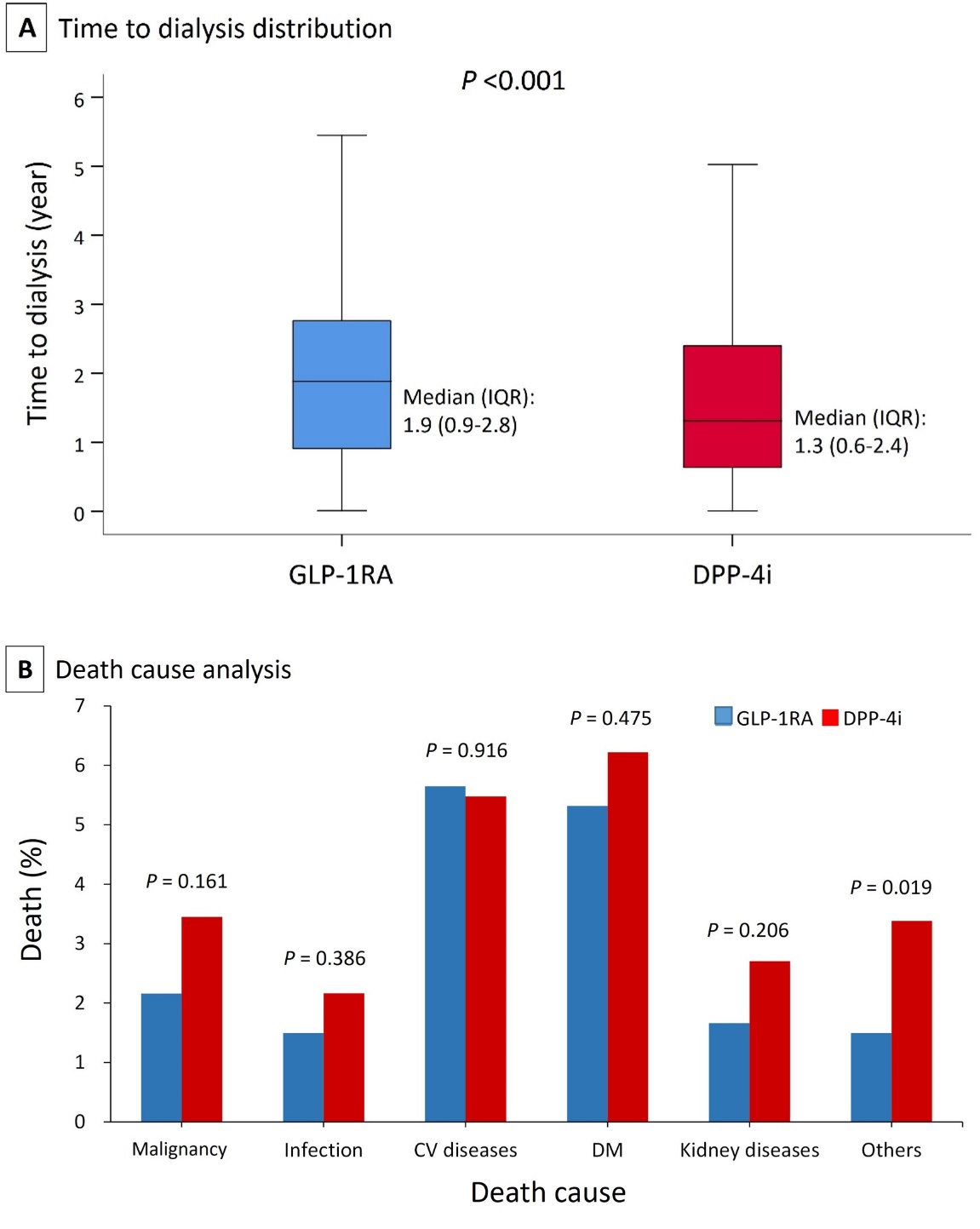


Time to dialysis was significantly reduced in the GLP-1RA group, with a 6-month delay compared to the DDP-4i group. Common causes death in the advanced DKD patients included malignancy, infection, CV diseases, DM, and kidney disease. There were no significant differences between the GLP-1RA and DPP-4i groups. The other causes of death were significantly lower in the GLP-1RA group.

Abbreviations: GLP-1RA, glucagon-like peptide 1 receptor agonist; DPP-4i, dipeptidyl peptidase 4 inhibitor; CV, cardiovascular; DM, diabetes mellitus.

**Table S1.** Number of patients with advanced chronic kidney disease with prescription of GLP1RA and DPP4i

| Class / agent | 2012 | 2013 | 2014 | 2015 | 2016 | 2017 | 2018 | 2019 | 2020 | 2021 |
| --- | --- | --- | --- | --- | --- | --- | --- | --- | --- | --- |
| DPP4i | 11,570 | 9,280 | 10,903 | 14,651 | 14,447 | 12,774 | 12,721 | 11,465 | 10,718 | 11,525 |
| Sitagliptin | 11,570 | 9,280 | 10,267 | 10,363 | 7,471 | 4,564 | 5,214 | 4,545 | 4,464 | 4,789 |
| Vildagliptin | 0 | 0 | 0 | 987 | 2,453 | 2,799 | 2,486 | 1,730 | 1,456 | 1,633 |
| Saxagliptin | 0 | 0 | 636 | 3,301 | 3,021 | 2,685 | 2,177 | 1,452 | 913 | 806 |
| Linagliptin | 0 | 0 | 0 | 0 | 1,502 | 2,726 | 2,844 | 3,526 | 3,590 | 3,855 |
| Alogliptin | 0 | 0 | 0 | 0 | 0 | 0 | 0 | 0 | 0 | 0 |
| GLP1RA | 0 | 0 | 20 | 204 | 375 | 231 | 244 | 660 | 1,118 | 1,532 |
| Exenatide | 0 | 0 | 20 | 204 | 35 | 8 | 8 | 0 | 0 | 0 |
| Liraglutide | 0 | 0 | 0 | 0 | 340 | 223 | 236 | 407 | 681 | 585 |
| Lixisenatide | 0 | 0 | 0 | 0 | 0 | 0 | 0 | 0 | 0 | 0 |
| Dulaglutide | 0 | 0 | 0 | 0 | 0 | 0 | 0 | 253 | 437 | 947 |
| Semaglutide | 0 | 0 | 0 | 0 | 0 | 0 | 0 | 0 | 0 | 0 |

Abbreviations: GLP1RA, glucagon-like peptide-1 receptor agonist; DPP4i, dipeptidyl peptidase 4 inhibitors;

Data were presented as frequency.

**Table S2.** Number of patients receiving dialysis with prescription of GLP1RA and DPP4i

| Class / agent | 2012 | 2013 | 2014 | 2015 | 2016 | 2017 | 2018 | 2019 | 2020 | 2021 |
| --- | --- | --- | --- | --- | --- | --- | --- | --- | --- | --- |
| DPP4i | 1,885 | 1,465 | 1,593 | 1,884 | 1,849 | 1,680 | 1,568 | 1,327 | 1,227 | 1,353 |
| Sitagliptin | 1,885 | 1,465 | 1,514 | 1,285 | 841 | 369 | 410 | 270 | 259 | 259 |
| Vildagliptin | 0 | 0 | 0 | 75 | 195 | 319 | 265 | 173 | 130 | 154 |
| Saxagliptin | 0 | 0 | 79 | 524 | 466 | 273 | 174 | 95 | 63 | 48 |
| Linagliptin | 0 | 0 | 0 | 0 | 347 | 719 | 719 | 774 | 758 | 873 |
| Alogliptin | 0 | 0 | 0 | 0 | 0 | 0 | 0 | 0 | 0 | 0 |
| GLP1RA | 0 | 0 | 2 | 30 | 29 | 27 | 17 | 56 | 121 | 174 |
| Exenatide | 0 | 0 | 2 | 30 | 6 | 1 | 1 | 0 | 0 | 0 |
| Liraglutide | 0 | 0 | 0 | 0 | 23 | 26 | 16 | 33 | 71 | 72 |
| Lixisenatide | 0 | 0 | 0 | 0 | 0 | 0 | 0 | 0 | 0 | 0 |
| Dulaglutide | 0 | 0 | 0 | 0 | 0 | 0 | 0 | 23 | 50 | 102 |
| Semaglutide | 0 | 0 | 0 | 0 | 0 | 0 | 0 | 0 | 0 | 0 |

Abbreviations: GLP1RA, glucagon-like peptide-1 receptor agonist; DPP4i, dipeptidyl peptidase 4 inhibitors;

Data were presented as frequency.
